# Evidence for positive selection in the gene *fruitless *in *Anastrepha *fruit flies

**DOI:** 10.1186/1471-2148-10-293

**Published:** 2010-09-24

**Authors:** Iderval S Sobrinho, Reinaldo A de Brito

**Affiliations:** 1 Universidade Federal de São Carlos, Departamento de Genética e Evolução, 13565-905. CP 676. São Carlos-SP. Brazil

## Abstract

**Background:**

Many genes involved in the sex determining cascade have indicated signals of positive selection and rapid evolution across different species. Even though *fruitless *is an important gene involved mostly in several aspects of male courtship behavior, the few studies so far have explained its high rates of evolution by relaxed selective constraints. This would indicate that a large portion of this gene has evolved neutrally, contrary to what has been observed for other genes in the sex cascade.

**Results:**

Here we test whether the *fruitless *gene has evolved neutrally or under positive selection in species of *Anastrepha *(Tephritidae: Diptera) using two different approaches, a long-term evolutionary analysis and a populational genetic data analysis. The first analysis was performed by using sequences of three species of *Anastrepha *and sequences from several species of *Drosophila *using the ratio of nonsynonymous to synonymous rates of evolution in PAML, which revealed that the *fru *region here studied has evolved by positive selection. Using Bayes Empirical Bayes we estimated that 16 sites located in the connecting region of the *fruitless *gene were evolving under positive selection. We also investigated for signs of this positive selection using populational data from 50 specimens from three species of *Anastrepha *from different localities in Brazil. The use of standard tests of selection and a new test that compares patterns of differential survival between synonymous and nonsynonymous in evolutionary time also provide evidence of positive selection across species and of a selective sweep for one of the species investigated.

**Conclusions:**

Our data indicate that the high diversification of *fru *connecting region in *Anastrepha *flies is due at least in part to positive selection, not merely as a consequence of relaxed selective constraint. These conclusions are based not only on the comparison of distantly related taxa that show long-term divergence time, but also on recently diverged lineages and suggest that episodes of adaptive evolution in *fru *may be related to sexual selection and/or conflict related to its involvement in male courtship behavior.

## Background

Several genes related to reproduction have shown higher rates of divergence than other genes in the genome [1,2]. This fast differentiation has been explained most often by positive selection mediated by sexual selection and/or sexual conflict [1,3,4], though some have suggested relaxed selective constraints [5,6]. Because these genes show high levels of divergence and may possibly be directly involved with reproductive isolation, some authors have suggested that such genes may be the best candidates to distinguish species that diverged recently and should actually be considered speciation genes [7,8].

**Figure 1:**
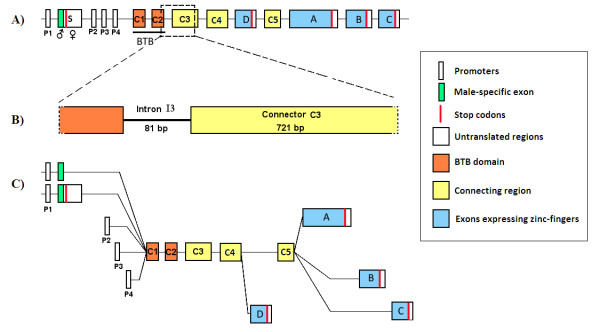
**Schematic representation of *fru *gene organization in *Drosophila***. A) Intron-exon organization of *fru *gene; P1 to P4 are alternative promoters; White boxes represent noncoding exons; S represents the sex-specifically spliced exon expressed only under P1 control; Orange boxes represent the BTB domain; Yellow boxes represent the connecting region; Boxes labeled A, B, C, and D represent alternative exons containing Zinc-finger domains (DNA-binding domains). B) Homologous region amplified in *Anastrepha*; Part of the BTB exon C2 was amplified but it was not used in the analyses. C) Alternative spliced transcripts. (Modified from Demir and Dickson [12])

Most evolutionary studies on genes related to sex differentiation have focused on a portion of those rapidly evolving genes involved in fertilization or male-female interaction and only a few on the genes responsible for sexual differentiation themselves. In Diptera, *transformer *(*tra*) and *doublesex *(*dsx*) are two of the main genes that control sexual differentiation that have been studied [5,9-11]. Another important gene in the sex-determination cascade is *fruitless *(*fru*), which controls the male courtship behavior by the establishment and development of a male-specific neuronal circuitry [12-14] along with *dsx *[15]. Its genomic organization is conserved in several lineages of insects [6,16] being composed of four main regions: sex-specific domain, dimerization domain (BTB), connecting region and DNA-binding domains (zinc-fingers) [12] (Figure 1A). The *fru *gene is alternatively spliced in a sex-dependent way from the P1 promoter, where the female transcript translation is interrupted by the binding of TRA/TRA-2 complex, whereas the male product is functional [17]. In sex-specific transcripts the exon (S) makes the first domain whereas, in non-sex-specific transcripts the first domain is composed by the BTB exons (Figure 1C). Depending on the species, the BTB domain is formed by one or two exons (C1-C2). In the same way the connecting region is formed by two to four exons depending on the taxonomic group (C3-C5). Finally, the DNA-binding domain at the 3' end is composed of one of four different exons (A-D), selected by differential splicing [6,16]. In addition to the sex-specific transcripts, sex-nonspecific products are expressed by three different promoters (P2, P3 and P4) downstream to the exon S, which confers to *fru *a complex pattern of expression in different tissues and stages of the development [18,19] (Figure 1C).

Considering that *fru *is one of the key elicitors of male courtship behavior, variation in its nucleotide sequence could be subjected to sexual selection and thus be involved in lineage isolation and differentiation. Should this be the case, we would expect a pattern of rapid divergence in *fru*, especially in regions under positive selection. Indeed, the comparison among different insect lineages shows that the N-terminal and the connecting region are the most divergent [6,16], which has been hypothesized to be either a consequence of these regions not being essential for the proper function of FRU protein, or because they would contain information for species-specific male courtship behavior [6]. Although the hypothesis of species-specific signature was rejected by the rescue of *fru *function *via *ectopic expression [6], a formal study of selection patterns on this gene has not yet been performed.

In this work we study patterns of genetic variability in *fru *in tephritid (Diptera) species of the group *fraterculus *to contrast the hypotheses that the high divergence of the connecting region is due to a relaxed selective constraint or to positive selection over the region. These *Anastrepha *species from the group *fraterculus *are generally identified by subtle differences in morphological traits, particularly the aculeus [20], which limits the identification to females. The problem of identifying species in this group is further enhanced by the inherent plasticity of the aculeus [21] and possible existence of many cryptic species in the group [22]. This problem has not been placated by the use of some molecular markers, such as *mtCOI *[23], *doublesex *[24] and *transformer *[25], which have, in general, shown low phylogenetic resolution. Therefore, even though we would like to test whether the gene *fru *would provide good genetic markers to discriminate species from the *fraterculus *group, notably *A. fraterculus*, *A. sororcula *and *A. obliqua*, we are mostly interested in determining the effects of selection in this region, which is non-trivial since we are contrasting species and populations that diverged recently, where the use of standard dN/dS methods [26-28] has been shown to be problematic [29]. Even though there is a plethora of neutrality tests already developed to detect departures from selection at the population level [30-32], the majority perform frequency analyses per nucleotide [30,31,33], and few have used haplotype information [34,35] or the phylogenetic relationships [36,37] as a framework to investigate patterns of departure from neutrality. Here, we propose a test that uses such information to detect patterns of selection using population polymorphism data rather than fixed differences among lineages.

## Results

### Long-term evolutionary analyses and positive selection in *fru*

**Figure 2:**
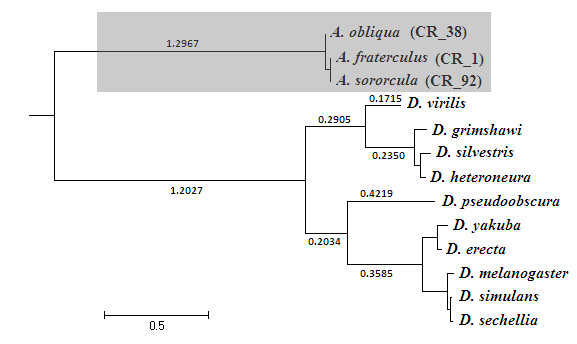
**ML phylogenetic tree of *fru *C3 exon used for detection of selection**. The *foreground *branch is highlighted in gray. The genetic distances are in nucleotide substitutions per codon.

For the long-term evolutionary study of the *fru *connecting region, we amplified a region of 802 bp containing an 81 bp intron (I3 intron) and 721 bp of the first exon from the connecting region (C3 exon) from three closely related *Anastrepha *species (*A. fraterculus*, *A. sororcula *and *A. obliqua*) (Figure 1B). The selective pressures over the *fru *C3 exon were investigated by the ratio of nonsynonymous to synonymous rates ratio. Table 1 shows the parameters inferred for the null models M1a and MA as well as for the alternative MA model. The relaxed branch-site test rejected the null model of selective constraint (0 < ω ≤ 1) indicating that the *foreground *branch (Figure 2) diverged in this region by relaxed selective constraint or by positive selection (Table 2). We contrasted the restricted MA versus MA models in the strict branch-site test to discriminate between the two hypotheses, and again the null model of selective constraint was rejected (Table 2) in favor of the hypothesis that part of the C3 exon differentiated by positive selection.

**Table 1:** Parameters estimates and log likelihood values for branch-site M1a, MA and restricted MA model.

Model	Parameters	Lnl
M1a *Relaxed branch-site test null model*	*ω*_0 _= 0.065 *p*_0 _= 0.870 *ω*_1 _= 1.000 *p*_1 _= 0.130	-3647.925
Restricted MA (*ω*_2 _= 1, fixed) *Strict branch-site test null model*	*ω*_0 _= 0.050 *ω*_2 _= 1.000 *p*_0 _= 0.653 *p*_1 _= 0.096 (*p*2 + *p*3) = 0.252	-3629.574
MA *Alternative model*	*ω*_0 _= 0.053 *ω*_2 _= 82.666 *p*_0 _= 0.679 *p*_1 _= 0.093 (*p*2 + *p*3) = 0,228	-3627.665

Lnl = log likelihood; *ω*_0 _= dN/dS values for sites with 0 <*ω *< 1; *ω*_1 _= dN/dS values fixed to 1; *ω*_2 _= dN/dS values for sites with *ω *> 1, which corresponds only to sites in the *foreground *branch.

**Table 2:** Comparison of null and alternative models by LRT and positively selected sites estimated by Bayes Empirical Bayes.

Test	Contrast	LRT	**D.F**.	χ^2^- Probability	Positively selected sites
Relaxed branch-site	M1a × MA	40.520	2	*p *< 10^-8^	16, 25, 31, 49, 61, 64, 65, 83, 105, 111, 131,
Strict branch-site	Restricted MA × MA	3.817	1	*p *< 0.05	164, 167, 188, 194, 195

LRT - likelihood ratio test; D.F - Degrees of freedom.

**Figure 3:**
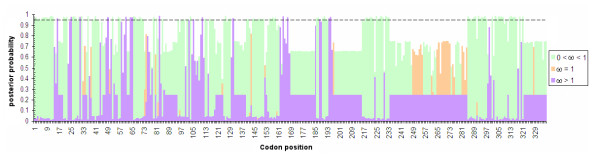
**Posterior probabilities for sites in *fru *C3 exon to evolve under purifying selection (0 <*ω *< 1); positive selection (*ω *> 1) and neutrality (*ω *= 1)**. The dashed line stands for 0.95 posterior probability limit.

**Figure 4:**
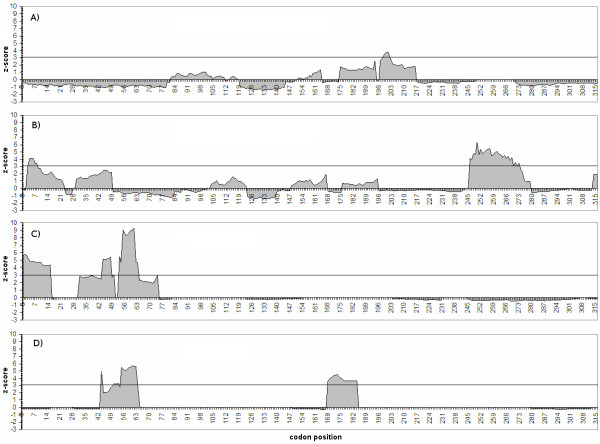
**Sliding window plots of the z-scores of radically changed properties showing regions under positive-destabilizing selection in *fru *C3 exon**. Dashed horizontal line indicates the Bonferroni corrected significant limit (z-score = 3.09, *p *< 0.001). A) α-helical tendencies - category 6 of radical change; B) α-helical tendencies - category 8 of radical change; C) Hydropathy - category 6 of radical change; D) Molecular weight category 6 of radical change.

Using Bayes Empirical Bayes we estimated that 16 sites were evolving under positive selection with posterior probability greater than 0.95 (Table 2; Figure 3). Figure 3 shows that positively selected sites are concentrated in the 5' half of the C3 exon, and that selectively constrained sites predominates in the terminal portion of the exon. The investigation of selection on amino acid properties in *TreeSAAP *analyses indicate that all 31 physicochemical properties examined showed significant departure from neutrality in the goodness-of-fit test, under a 0.05 cutoff limit (Table 3). Nevertheless, only three properties had significantly positive z-scores under the radical changes categories (6 to 8): α-helical tendencies (*Pα*); Hydropathy (*H*) and Molecular weight (*Mw*). The sliding window analysis in *TreeSAAP *also indicates positive-destabilizing selection in the 5' end of C3 exon (Figure 4). These macroevolutionary contrasts indicate presence of positive selection at the 5' end of C3 exon, whereas there is purifying selection at the 3' end of this exon.

**Table 3:** Amino acid physicochemical properties under positive destabilizing selection in *fru *C3 exon.

Physicochemical property	Goodness-of-fit (neutral expectation)	radical change category (6, 7 e 8)	z-score
Alpha-helical tendencies (*P_α_*)	36.498***	8	1.975*
Average number of surrounding residues (*N_s_*)	76.820***	-	-
Beta-structure tendencies (*P_β_*)	67.631***	-	-
Bulkiness (*B_l_*)	40.169***	-	-
Buriedness (*B_r_*)	47.688***	-	-
Chromatographic index (*R_f_*)	65.981***	-	-
Coil tendencies (*P_c_*)	82.908***	-	-
Composition (*C*)	52.020***	-	-
Compressibility (*K*^0^)	36.874***	-	-
Equilibrium constant (ionization of COOH) (*pK'*)	33.814***	-	-
Helical contact area (*C_a_*)	41.651***	-	-
Hydropathy (*H*)	92.128***	6	4.972***
Isoelectric point (*pH_i_*)	56.298***	-	-
Long-range non-bonded energy (*E_l_*)	38.021***	-	-
Mean r.m.s. fluctuation displacement (*F*)	57.826***	-	-
Molecular volume (*M_v_*)	48.275***	-	-
Molecular weight (*M_w_*)	53.230***	6	3.928***
Normalized consensus hydrophobicity (*H_nc_*)	29.042***	-	-
Partial specific volume (*V*^0^)	22.173**	-	-
Polar requirement (*P_r_*)	77.854***	-	-
Polarity (*P*)	117.046***	-	-
Power to be at the C-terminal of alpha-helix (*α_c_*)	57.177***	-	-
Power to be at the middle of alpha-helix (*α_m_*)	40.309***	-	-
Power to be at the N-terminal of alpha-helix (*α_n_*)	35.782***	-	-
Refractive index (*μ*)	17.263*	-	-
Short and medium range non-bonded energy (*E_sm_*)	73.460***	-	-
Solvent accessible reduction ratio (*R_a_*)	16.175*	-	-
Surrounding hydrophobicity (*H_p_*)	90.284***	-	-
Thermodynamic transfer hydrophobicity (*H_t_*)	90.401***	-	-
Total non-bonded energy (*E_t_*)	32.042***	-	-
Turn tendencies (*P_t_*)	45.706***	-	-

* *p *< 0.05; ** *p *< 0.01; *** *p *< 0.001

### Analysis of Positive Selection at the Population Level

Table 4 summarizes estimates of nucleotide variability for the region sequenced (I3 intron and C3 exon). Considering only the C3 exon of the 50 individuals sequenced from the three species, 167 polymorphic sites were found, nine with more than one nucleotide variant, violating the infinite allele model. The I3 intron had 28 polymorphic sites, six of them with more than one nucleotide variant (Table 4). The comparison among sequences also revealed 38 different intron haplotypes and 94 C3 exon haplotypes, with the majority composed by unique haplotypes, revealed by the high haplotype diversity levels both for intron and exon. Diversity indexes for the coding regions show that synonymous nucleotide diversities (*π*_s_) are greater than nonsynonymous nucleotide diversities (*π*_a_) (z-values for all *π*_a × _*π*_s _comparisons were lower than -30, *p *< 0.001) (Table 4). Because recombination may affect nucleotide diversity and may destroy historical information in a region, we used the software *RDP*, which failed to detect any significant signal of recombination.

**Table 4:** Genetic diversity estimates in the *fruitless *C3 exon and intron.

	Species	N	h	*Hd* (SD)	S	S_y_	N_Sy_	sg	m_3_	*π* (SD)	*π*_s_ (SD)	*π*_a_ (SD)	*θ* (SD)
	*A. fraterculus*	37	36	0.998 (0.007)	82	42	34	78	4	0.011 (0.0005)	0.027 (0.0021)	0.006 (0.0010)	0.028 (0.009)
Exon	*A. obliqua*	41	40	0.999 (0.006)	80	41	39	77	3	0.013 (0.0005)	0.026 (0.0020)	0.008 (0.0007)	0.027 (0.008)
	*A. sororcula*	19	19	1.000 (0.017)	62	39	26	60	2	0.014 (0.0005)	0.034 (0.0031)	0.007 (0.0011)	0.025 (0.009)
	*Anastrepha *sp.	97	94	0.999 (0.002)	167	83	78	157	9	0.013 (0.0005)	0.030 (0.0015)	0.007 (0.0004)	0.047 (0.009)

	*A. fraterculus*	37	22	0.922 (0.030)	17	-	-	17	0	0.027 (0.0022)	-	-	0.057 (0.021)
Intron	*A. obliqua*	41	11	0.429 (0.097)	8	-	-	8	0	0.009 (0.0024)	-	-	0.026 (0.012)
	*A. sororcula*	19	13	0.936 (0.037)	16	-	-	15	1	0.039 (0.0058)	-	-	0.064 (0.026)
	*Anastrepha *sp.	97	38	0.834 (0.032)	28	-	-	22	6	0.026 (0.0022)	-	-	0.077 (0.023)

N = number of sequences; h = number of haplotypes; *Hd *= haplotype diversity; S = Number of polymorphic sites; S_y _= number of synonymous changes; N_Sy _= number of nonsynonymous changes; sg = singletons; m_3 _= sites with more than three variants; *π *= nucleotide diversity; *π*_s _= synonymous nucleotide diversity; *π*_a _= nonsynonymous nucleotide diversity; *θ *= Waterson's theta. SD = standard deviation.

**Figure 5:**
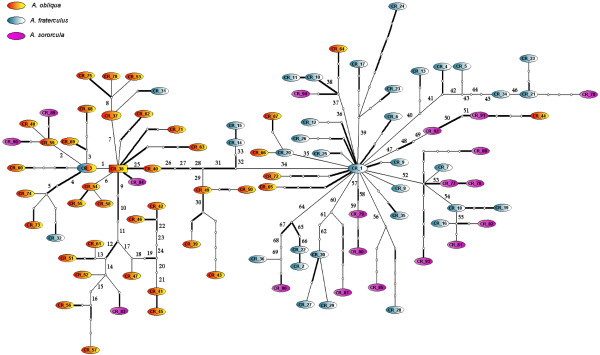
**Haplotype network of *fru *C3 exon**. Empty circles indicate missing intermediate haplotypes (extinct or unrepresented in the sample). Each line indicates a mutation step which separates different haplotypes. The area of the circles representing each haplotype is proportional to the number of individuals carrying the haplotype.

**Figure 6:**
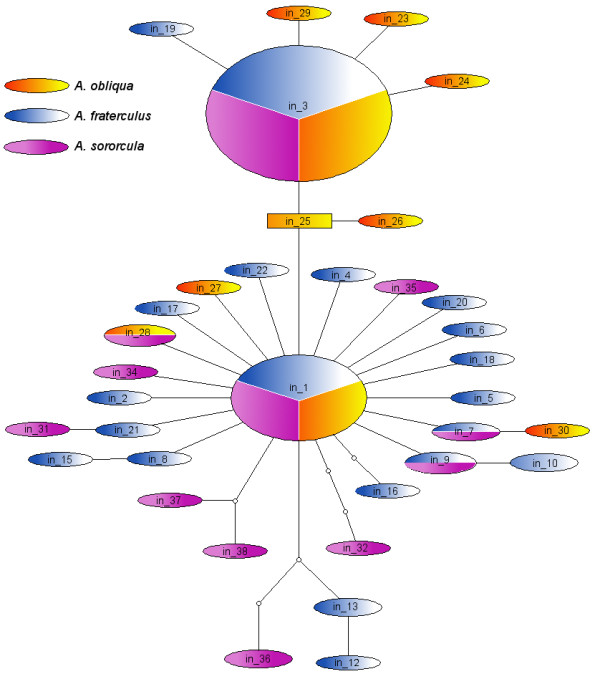
**Haplotype network of *fru *intron**. Empty circles indicate missing intermediate haplotypes (extinct or unrepresented in the sample). Each line indicates a mutation step which separates different haplotypes. The area of the circles representing each haplotype is proportional to the number of individuals carrying the haplotype.

We performed several neutrality tests on the populational data separated by species. The analyses on the exon of *A. obliqua *revealed that all tests were significantly negative, whereas only Fay and Wu's *H *and Tajima's *D *were significantly negative for *A. fraterculus *and *A. sororcula*. On the other hand, when these tests were performed on the intron data, we only got significant departures from neutrality in *A. obliqua *for the Tajima's *D *test and Fay and Wu's *H *and in *A. fraterculus *for the Fay and Wu's *H *test (Table 5). We used statistical parsimony to establish haplotype networks for the C3 exon (Figure 5) and for the I3 intron (Figure 6). Because these haplotype networks fail to indicate long branches separating the different species here studied, we also performed the neutrality tests combining all sequences in a single set. When we do so, all neutrality tests performed either on the intron or on the exon data are significantly negative (Table 5).

**Table 5:** Neutrality tests of C3 exon and intron.

	Species	Fu and Li's *D*	Fu and Li's *F*	Fay and Wu's *H*	Tajima's *D*
	*A. fraterculus*	-1.584 ^a^	-2.197	-46.276**	-2.244**
Exon	*A. obliqua*	-2.355*	-2.590*	-30.660**	-1.924*
	*A. sororcula*	-1.596 ^a^	-2.038^a^	-24.883**	-1.882*
	*Anastrepha *sp.	-4.578*	-4.334*	-81.514**	-2.435**

	*A. fraterculus*	-1.951^a^	-2.157^a^	-4.532*	-1.739^a^
Intron	*A. obliqua*	-1.181	-1.389	-2.813*	-1.839*
	*A. sororcula*	-1.205	-1.497	-2.164	-1.476
	*Anastrepha *sp.	-3.528*	-3.412*	-8.766*	-1.989*

* *p *< 0.05; ** *p *< 0.01; a - not significant (0.10 >*p *> 0.05)

The topology of the haplotype network was then used to compare the probability of survival through time of synonymous and nonsynonymous mutations by estimating the number of haplotypes defined by each type of substitution. We observed 86 tip nonsynonymous mutations to 93 tip synonymous mutations, and 23 interior nonsynonymous to 44 synonymous mutations. The data from *fru *gene in *Anastrepha *(Table 6) indicate that, in spite of their smaller number, nonsynonymous mutations define, on average, haplotypes with higher frequencies than those defined by synonymous mutations. This is indicated by a significant two-tailed Mann-Whitney ranking test (*U *= 106; *p *< 0.02), in which we observe that nonsynonymous mutations have a mean rank of 29.7 and mean number of derived haplotypes of 27.8, whereas synonymous substitutions have a mean rank of 22.7 and mean number of derived haplotypes of 6.2.

**Table 6:** Frequency-based ranking of synonymous and nonsynonymous mutations.

Mutation type	Internal mutation	f*	Ranking (tied)
Synonymous	6	3	7.5
Synonymous	8	3	7.5
Synonymous	35	3	7.5
Synonymous	39	3	7.5
Synonymous	46	3	7.5
Synonymous	47	3	7.5
Synonymous	49	3	7.5
Synonymous	55	3	7.5
Synonymous	61	3	7.5
Synonymous	62	3	7.5
Synonymous	63	3	7.5
Synonymous	4	3	7.5
Synonymous	5	3	7.5
Synonymous	48	3	7.5
Synonymous	2	4	20.5
Synonymous	14	4	20.5
Synonymous	18	4	20.5
Synonymous	29	4	20.5
Synonymous	36	4	20.5
Synonymous	37	4	20.5
Synonymous	43	4	20.5
Synonymous	44	4	20.5
Synonymous	45	4	20.5
Synonymous	60	4	20.5
Synonymous	64	4	20.5
Nonsynonymous	19	4	20.5
Synonymous	17	5	28
Synonymous	42	5	28
Synonymous	54	5	28
Synonymous	7	6	31
Synonymous	41	6	31
Nonsynonymous	12	6	31
Synonymous	40	7	33
Synonymous	52	10	34
Synonymous	11	11	36
Nonsynonymous	9	11	36
Nonsynonymous	10	11	36
Synonymous	1	12	38
Synonymous	34	53	39
Nonsynonymous	31	55	40
Nonsynonymous	26	59	42
Nonsynonymous	27	59	42
Nonsynonymous	28	59	42
Nonsynonymous	25	60	44

f* - Number of haplotypes defined by the mutation.

## Discussion

### Positive selection in *fru *C3 exon

Comparisons of *fru *sequences from insects of very distinct evolutionary lineages show that regardless of the high evolutionary conservation of the BTB and DNA-binding domains, the connecting region show high divergence across the different species studied [6,16]. These results are compatible with relaxed selective constraint on the latter portion of the gene or with divergence by positive selection. Here we contrast these two hypotheses using both, population and species level data from three species of *Anastrepha *to show that in all levels our data are better explained by positive selection acting on specific portions of the gene, rather than relaxed constraint in the connecting region (C3 exon).

The combination of the relaxed branch-site test (M1a *vs*. MA models) (Table 2) and the strict branch-site test (restricted MA *vs*. MA) demonstrates that part of the differentiation in the connecting region C3 exon from *fru *between the *background *(Drosophilidae) and *foreground *(Tephritidae) lineages is due to positive selection (Table 2). A BEB analysis detected at least 16 sites under positive selection, 14 of which were inserted in regions where positive-destabilizing selection was inferred by the MM01 analysis (Table 3, Figure 3 and Figure 4)

Because the BTB domain and the DNA-binding domains are generally conserved while the connecting region is highly divergent, it was suggested that the connecting region would either play no important role in FRU function or it would contain species-specific information. According to the first hypothesis, the higher divergence of the connecting region would be due to relaxed selective constraint or neutral evolution, whereas according to the second hypothesis its variation would be explained by adaptive selection. Gailey *et al. *[6] rejected this latter hypothesis because the transgenic expression of *Anopheles fru *in *D. melanogaster *rescued the Muscle of Lawrence (MOL) development, a structure that is specified only by male Fru^M ^protein, which would imply that relaxed selective constraint or neutral evolution should be invoked to explain its variation. However, our studies on selective pattern in *fru *C3 exon from connecting region show signals of adaptive evolution in this region.

The gene *fru *is the most complex in terms of genomic organization, when compared to the other main genes from sex-determination cascade (*doublesex *and *transformer*). Due to alternative splicing, *fru *may be expressed in at least four different male isoforms and four non-sex-specific isoforms [12] (Figure 1C). The combination of these isoforms is required for the correct development of some neuronal circuitry and connections necessary to control courtship and copulation behavior [38] All these isoforms share four exons that are commonly expressed that include the BTB region and the connecting region. We sequenced a large portion of the latter region, which may be postulated to help connecting the BTB to the zinc fingers present at the end of each of the alternative spliced isoforms. The interactions among these isoforms and the involvement of FRU proteins in features directly and indirectly subject to sexual selection offer opportunities for positive selection to occur over some segments of this gene. These several distinct aspects of sexual differentiation could be affected by the connecting region differently than the MOL development, hence the rescue of the latter does not mean that other subtler aspects of the behavior effected by *fru *such as male courtship song [39], response to sex-pheromones [40] and female post-copulatory behavior [41], would respond similarly. This pattern is akin to what has been described for some central genes in gene networks which are co-opted and assume new functions in different developmental contexts [42]. Such patterns have been observed in several developmental genes, such as some HOX genes, which affect basic pattern formation as well as wing and other appendages formation in arthropods [43,44]. It should be mentioned, though, that caution should be taken when considering sexual selection as a sole explanation for the positive selection here found, because *fru *also codes for non-sex-specific transcripts [12], so it is possible that the signal of adaptive selection may be the result of selection over phenotypes not related to sexual behavior.

### Selective pattern in low divergence lineages of *Anastrepha*

Both dN/dS and MM01 analyses require that the differences among sequences represent fixed substitutions among very well defined lineages [29,45,46]. When dealing with data from recently diverged lineages, few fixed substitution are found among sequences, and consequently, the power of the tests to discriminate between positive and purifying selection is reduced [47]. Additionally, recently diverged lineages, such as the species *A. fraterculus*, *A. sororcula *and *A. obliqua *here considered, may still segregate ancestral polymorphisms. For this reason, standard interpretation of dN/dS and MM01 statistics could lead to equivocal conclusions and other approaches are required.

Our data cannot be subject to the McDonnald-Kreitman [36] test since there is no fixed interspecific variation for any of the species considered. We may, however, use the same rationale of contrasting recent and old synonymous and nonsynonymous mutations in the framework of the haplotype network. The McDonnald-Kreitman test considers two categories of contrasts, polymorphic and fixed, or recent and old, but there is more information available from the network, particularly the relative frequency of each mutation, which may be an indication of the relative age of a mutation [48]. In a neutrally evolving region, older mutations would tend to be in higher frequencies than new mutations [49], that is why we performed a contrast between synonymous and non-synonymous mutations using a non-parametric Mann-Whitney test which investigated the pattern of differential survival between synonymous and nonsynonymous in evolutionary time (Table 6). This Mann-Whitney test rejects the null hypothesis that synonymous and nonsynonymous mutations come from the same distribution, and indicates that nonsynonymous mutations survived longer than synonymous mutations (*p *< 0.02). The excess of low-frequency variants could be explained by many other factor besides positive selection, such as population demographic change and background selection, however, only positive selection and very specific demographic scenarios are able to explain the excess of high frequency mutations [32]. Then the excess of nonsynonymous mutations found in the *fru *C3 exon reveals the action of positive selection driving the increase in frequency of such mutations in the C3 exon. Because this test is dependent on the topology of the haplotype network, which has been shown to vary due to stochastic processes [50], we are currently evaluating the power of this test in different evolutionary scenarios using forward and reverse-time simulations, which will be the dealt with elsewhere.

The excess of high frequency mutations detected in the Mann-Whitney test was also detected by the significantly negative Fay and Wu's *H *which measures the frequency of high derived mutations (Table 5). The Tajima's *D *and the Fu and Li's *D *statistics were also significantly negative and indicate an excess of low frequency variants. The joint analysis of different neutrality tests allows for a better evaluation of the influence of selection over this region, since each one is sensitive to a particular aspect of the site frequency spectrum [51]. One evident signal of hitchhiking effect by positive selection is the excess of low and high frequency variants coexisting in the a population after many generations from the selective sweep [32], while the expected pattern for purifying selection would be only the excess of low frequency variants. Therefore, the joint analysis of these neutrality tests confirms that *fru *C3 exon from *Anastrepha *shows signals of positive selection, if not in the region itself, in a closely linked region.

### Selective sweep in *fru *connecting region

The diversity indexes of the C3 exon are significantly lower than those of the I3 intron (all z-values < -5, *p *< 0.001) (Table 4), which would suggest that the intron has been evolving in a less constrained way. This is not surprising since it is expected that non-coding regions, such as introns, would evolve neutrally, though some introns have been described to evolve more conservatively than adjacent coding regions [52]. Interestingly, when we partition the diversity estimates by species we observe a reduction in the diversity levels for the intron in *A. obliqua*, but not in the adjacent exon. In *A. obliqua*, π and θ values for the intron are three times lower than those estimated for *A. fraterculus *and *A. sororcula *(z-values < -4, *p *< 0.001) (Table 4). Even the haplotype diversity was twice smaller than the values for the other two species (z-values < -4, *p *< 0.001). If we consider that the intron has evolved neutrally, we would expect similar diversity levels for the three species. Departures from these expectations would suggest that populations have experienced distinct demographic scenarios or different selection events have acted upon the intron or contiguous regions. Because the reduction in intron diversity in *A. obliqua *was not observed in the contiguous connecting region, it is not likely that it was caused by a recent demographic event, otherwise it should have affected equally the genetic variation in the intron and the coding region [53,54]. On the other hand, a selective sweep would lead to a reduction in local genetic variation with increase in frequency of the few polymorphisms associated with the sweep, and depending on its intensity could lead to complete fixation in the region [54]. When only drift and mutation are contributing to the increase of the diversity after such reduction, it takes on average 4*N *generations for the diversity to reach Fisher-Wright equilibrium levels. If there is purifying selection it would take longer for the equilibrium to be reached, whereas when there is positive selection the favored alleles, or others linked to them, will rapidly recover their high allele frequencies. Therefore, the existence of a previous selective sweep should have longer lasting effects on the genetic variation of neutral regions than on regions under positive selection. The similar genetic variability in the coding region of the three species, coupled with a reduction in the variation in *A. obliqua *intron, suggests a selective sweep in *A. obliqua*, from which the intron variation is still recovering, while the variation in the coding region, subjected to positive selection, has already recovered, or was never completely lost. This scenario is more likely if more than one site has been subject to positive selection at the same time [55], as it seems to be the case here. The hypothesis of a recent selective sweep is also corroborated by a star-like haplotype network with an excess of rare haplotypes for the intron, and lower Fay and Wu's *H *estimates for the coding region than for the intron (Figure 6).

### *fru *as species-specific marker

The lineage sorting of ancestral polymorphisms makes recently diverged species share alleles throughout their genome causing a conflict between gene tree and species tree for several genes or DNA segments [56]. When only neutral markers are considered it is expected that most of the loci attain reciprocal monophyly only after 9*N*_*e *_generations from the speciation event [57,58], which would take a long time if the species have large effective sizes. Considering that *Anastrepha *species from the fraterculus group have diverged recently [23,59] and should have large effective population sizes, we expect that species in this group will still show high degree of shared ancestral polymorphisms throughout their genome, which has been suggested by previous studies using both mitochondrial and nuclear genes [23,25,60]. Even though we did not observe strict reciprocal monophyly when using data from *fru*, most specimens of *A. obliqua *are separated from the other species by a branch with several mutations, mostly amino acid replacements. In fact, only five haplotypes of this branch belong to specimens that were diagnosed as *A. fraterculus *and four as *A. sororcula *(Figure 5). When studying genes directly involved in the species reproductive isolation, the ancestral polymorphisms associated with regions under selection would be wiped away at a faster rate, and consequently, one or both diverging groups would be fixed for species-specific variation at that gene before other genome regions [61]. Multilocus studies of closely related species have reported extensive ancestral polymorphisms sharing, but exclusive variation in some genes related to reproductive traits such as pheromone production [62], seminal proteins [7,63] or spermatogenic function [61]. It is possible that the fast evolutionary rate in *fru *may explain, *per se*, its more accurate phylogenetic resolution in *Anastrepha *species, but it may also be due to the fact that this gene is adaptively diverging, and has a role in determining courtship behavior, which could somehow affect reproductive isolation.

## Conclusions

Contrary to Gailey *et al. *[6], who had considered the high diversification of *fru *connecting region solely as a consequence of relaxed selective constraint, here we conclude that part of such diversification is due to positive selection. These conclusions are based not only on the comparison of distantly related taxa that show long-term divergence time, but also on recently diverged lineages and suggest that the episodes of adaptive evolution in *fru *may be related to sexual selection and/or conflict related to its involvement in male courtship behavior. Because the findings of an association between *fru *variation and the isolation of *A. obliqua *may only be because they had occurred historically concurrently, we need a more detailed study that considers the entire *fru *gene, as well as its interaction with other genes from the sexual differentiation cascade in more species to better investigate the role of the *fru *gene in the differentiation of this group and others.

## Methods

Fruits from different plant species that are known to be infested by *Anastrepha *were collected from 33 localities in Brazil (Table 7) and set in vermiculite for 14 days when pupae were separated. After emergence and maturation, flies that were identified as belonging to the *A. fraterculus *group, mostly *A. fraterculus*, *A. obliqua *and *A. sororcula*, were separated and immediately processed or preserved in 95% ethanol until DNA extraction.

**Table 7:** Sampling locations with geographic, haplotype and species information.

Code	Collection locality	Latitude (S)	Longitude (W)	Haplotype
01	São Carlos - SP	22° 01' 03''	47° 53' 27''	1-f.1, 2-f.1
02	Vitória - ES	20° 19' 10''	40° 20' 16''	38-o.2, 39-o.2, 72-o.2, 73-o.2
03	Feira do Santana -BA	12° 16' 00''	38° 58' 00''	69-o.3, 70-o.3
04	Gurupi - TO	11° 43' 45''	49° 04' 07''	57-o.4, 58-o.4
05	São Sebastião - SP	23° 45' 36''	45° 24' 35''	8-f.5, 9-f.5, 82-s.5, 83-s.5
06	Bertioga - SP	23° 51' 16''	46° 08' 19''	20-f.6, 21-f.6
07	Conceição do Almeida - BA	12° 46' 46''	39° 10' 12''	28-f.7, 29-f.7, 30-f.7, 31-f.7
08	Araguaína - TO	07° 11' 28''	48° 12' 26''	40-o.8, 41-o.8, 42-o.8, 43-o.8, 44-o.8, 45-o.8, 46-o.8, 47-o.8
09	Santo Amaro - BA	12° 32' 48''	38° 42' 43''	1-f.9, 27-f.9, 65-o.9, 66-o.9, 67-o.9, 68-o.9
10	Goiânia - GO	16° 40' 43''	49° 15' 14''	50-o.10, 51-o.10
11	Bonito - PE	08° 28' 13''	35° 43' 43''	3-f.11, 4-f.11, 5-f.11, 77-s.11, 78-s.11, 79-s.11
12	Redenção - PA	08° 01' 43''	50° 01' 53''	52-o.12, 53-o.12, 54-o.12
13	Babaçulândia - TO	07° 12' 17''	47° 45' 25''	38-o.13, 3-o.13
14	Pirenópolis - GO	15° 51' 09''	48° 57' 33''	55-o.14, 56-o.14
15	Três Lagoas - MS	20° 45' 04''	51° 40' 42''	61-o.15, 62-o.15
16	Belém -PA	01° 27' 21''	48° 30' 16''	63-o.16, 64-o.16
18	Belo Horizonte - MG	19° 55' 15''	43° 56' 16''	71-o.18
19	Bela Vista de Goiás - GO	16° 58' 22''	48° 57' 12''	48-o.19, 49-o.19
20	Linhares - ES	19° 23' 28''	40° 04' 20''	74-o.20, 75-o.20, 94-s.20, 95-s.20
21	Piracicaba - SP	22° 43' 31''	47° 38' 57''	84-s.21, 85-s.21, 86-s.21, 87-s.21
22	Nova Souré - BA	11° 14' 00''	38° 29' 00''	92-s.22, 93-s.22
23	Bauru - SP	22° 18' 53''	49° 03' 38''	24-f.23, 25-f.23, 88-s.23, 89-s.23, 90-s.23, 91-s.23
24	Porto Seguro - BA	16° 26' 59''	39° 03' 53''	80-s.24, 81-s.24
25	Boracéia - SP	22° 11' 35''	48° 46' 44''	10-f.25, 11-f.25
26	Bariri - SP	22° 04' 28''	48° 44' 25''	12-f.26, 13-f.26
27	Itabira - MG	19° 37' 09''	43° 13' 37''	6-f.27, 7-f.27
28	Moji das Cruzes - SP	23° 31' 22''	46° 11' 18''	22-f.28, 23-f.28
29	Vacaria - RS	28° 30' 44''	50° 56' 02''	32-f.29, 33-f.29, 34-f.29, 35-f.29
30	Vargem Alta - ES	20° 40' 17''	41° 00' 25''	16-f.30, 17-f.30, 18-f.30, 19-f.30
31	Natal - RN	05° 47' 42''	35° 12' 34''	14-f.31, 15-f.31
32	Caçador - SC	26° 46' 31''	51° 00' 54''	36-f.32, 37-f.32
33	Santa Isabel - SP	23°18' 0"	46°13' 0"	76-o.33

The numbers in haplotypes represent their identification and the letters represent the species at which they were found: o - *A. obliqua*; f - *A. fraterculus*; s - *A. sororcula*. Numbers after the letter stand for collection locality code.

### DNA extraction and sequencing

DNA was extracted following the modified protocol of [64], in which the exoskeletons were maintained intact for future morphological analyses. We amplified a region from the end of the BTB domain to next the end of first exon of the connecting region of *fru *(C3 exon) (Figure 1B), using degenerate primers created from homologous sequences of closely related species: (5'-AGTTCGCTGCCGATGTTYCTCAA-3' and 5'-GACAGRCACTAYCCGCAGGACTCTCAG-3'). This region was amplified by PCR from genomic DNA in a thermocycler PTC-200 (BioRad) using an admixture of *Taq *polymerase and *Pfu *polymerase to reduce incorporating errors [65]. PCR products were purified by PEG 8000 precipitation [66] and cloned with InsTAclone kit (Fermentas). At least two recombinant colonies were sequenced with forward and reverse M13 primers using the DYEnamic™ET dye terminator kit (GE Healthcare) and resolved either in a MegaBace 1000 (GE Healthcare) or in an ABI 3730 (Applied). DNA sequencing was mostly carried out at MACROGEN INC, Korea. Quality of base-calling was visually inspected in *Chromas *version 2.31 http://www.technelysium.com.au. The GenBank accession numbers for the 97 sequences from *A. fraterculus*, *A. sororcula *and *A. obliqua *are [HQ003715 - HQ003811. We used the translation of these *Anastrepha *exon sequences to proteins to align this region to more distantly related taxa. The protein alignments were used as reference to correct alignments of nucleotide sequences, which were used in the phylogenetic tree estimation. Sequences were aligned and visually inspected using *Clustal W *in *BioEdit Sequence Alignment Editor *software [67]. When sequenced clones from an individual differed by less than 3 mutations, additional recombinant colonies (up to five total) were sequenced to confirm results.

### *fruitless *evolution

We used a hierarchical strategy to test for selection on *fru *sequences. First we evaluated patterns of long-term evolutionary response to selection (i. e., a deeper phylogenetic level), by contrasting a sample of sequences from *Anastrepha *species against sequences from other Muscomorpha. We also tested for patterns of selection which may be detected at the population level, contrasting *fru *sequences from the connecting region and preceding intron gathered from *Anastrepha *collected from several localities in Brazil.

### Phylogenetic tree reconstruction

To reconstruct the phylogenetic tree an optimal nucleotide substitution model was determined by Akaike information criterion (AIC) using *MODELTEST *ver. 3.7 [68] implemented in the *HyPHy *package ver 0.95 beta [69]. A phylogenetic tree using sequences from *fru *C3 exon was estimated by maximum likelihood using the software *PhyML *ver. 3.0 [70] under the TIM+G nucleotide substitution model estimated previously. For the phylogenetic tree reconstruction we used one sequence from each of the *Anastrepha *species studied in this work (*A. fraterculus *[GenBank accession number: HQ003715], *A. sororcula *[GenBank accession number: 1376936] and *A. obliqua *[GenBank accession number: HQ003765]) to represent the Tephritidae lineage, and 10 different sequences from Drosophilidae available in GenBank: *D. simulans *- [AF297054.1]; *D. sechellia *- [AF297055.1]; *D. melanogaster *- [D84437.1]; *D. erecta *- [AF298222.1]; *D. yakuba *- [AF297056.1]; *D. heteroneura *- [AF051668.1]; *D. silvestris *- [AF051665.1]; *D. grimshawi *- [AF105124.1]; *D. virilis *- [AY028967.1] and *D. pseudoobscura *- [AF297059.1].

### Long-term response to selection in *fruitless*

In order to investigate selective pressures that modeled the evolution of *fru*, we performed a relaxed branch-site test and a strict branch-site test [71,72] using the software CODEML, implemented in PAML ver. 4 [73]. The nonsynonymous/synonymous substitutions rate ratios (dN/dS = ω) were measured to infer the selective pressure at the protein level. A ω > 1 at a specific site would indicate positive selection, because nonsynonymous substitutions would have higher fixation probabilities than synonymous mutations due to selective advantages. On the other hand, a ω < 1 would indicate purifying selection, caused by selective constraints at the codon position. The branch-site tests consider the phylogenetic tree to test different selective scenarios. The phylogenetic tree was separated in *foreground *branches, at which positive selection is tested, and *background *branches, represented by the other lineages. We established the *Anastrepha *branch as *foreground *and the other *Drosophila *species as *background*. Positive selection was inferred by a contrast of hypotheses using a maximum likelihood approach. Both relaxed branch-site test and strict branch-site test use the MA model as alternative model, in which the codons in the *foreground *were allowed to have ω > 1, and the codons in *background *were constrained to ω ≤ 1. The relaxed branch-site test null model (M1a) assumes the same evolutionary rates for all sites and branches, with all sites varying ω values from 0 and 1. On the other hand, the strict branch-site test fixes the ω > 1 category to 1 in the null model (restricted MA), that is, all sites with ω > 1 are forced to evolve neutrally (ω = 1). Positive selection is inferred when a log likelihood ratio test (LRT) of these values results in a significant value. To assess significance of the LRT we used a chi-squared null distribution composed of a mixture of point mass 0 and $\chi_{\text{1}}^{\text{2}}$. Under such null distribution the critical values at 0.05 and 0.01 levels were 2.71 and 5.41, respectively [71]. Bayes Empirical Bayes [28] method was used in conjunction with the branch-site test to estimate which sites were under the influence of positive selection. We used the MA model parameters to estimate the Bayes Empirical Bayes posterior probabilities. Because some models are prone to show a problem of lack of convergence in a likelihood framework, we ran the analyses twice with different initial ω values.

Positive selection was also investigated through the MM01 method of McClellan *et al. *[46] which evaluates whether nonsynonymous substitutions favored or not structural or functional changes in the protein. The analyses were carried out in *TreeSAAP *version 3.2 [46,74,75] and considered the changes in many physicochemical properties brought forth by each nonsynonymous substitution. A global deviation from neutrality is verified by a goodness-of-fit test between a neutral expected distribution and the observed distribution of the selected physicochemical properties [46]. Furthermore, *TreeSAAP *also separates the magnitude of nonsynonymous changes in a range going from conservative to very radical substitutions, according to the change in specific physicochemical properties. The lowest classes (1 to 3) represent the more conservative changes and the highest classes (6 to 8) represent the more radical changes [46]. McClellan *et al. *[46] conservatively defined stabilizing selection as a selection that tends to maintain the original biochemical attributes of the protein, despite the fact of the inference of positive selection, and destabilizing selection as a selection that favors structural and functional shifts in a region of a protein. In this way, positive-destabilizing selection represents a signature of molecular adaptation.

We considered 8 categories of magnitude change for the analysis and followed the categorization given in McClellan *et al. *[46]. Only amino acid properties identified by significant positive z-score in the magnitude categories 6, 7 or 8 were considered to be affected by positive-destabilizing selection. To verify which specific regions were affected by positive-destabilizing selection, we performed a sliding window analysis using the amino acid properties which were significant for this type of change. Sliding windows of 20 amino acid length with a sliding step of one codon were selected for showing the best signal-to-noise ratio [45].

### Genetic diversity and population evolutionary analyses

General diversity indexes, such as haplotype (*Hd*) and nucleotide (π) diversity [76], number of polymorphic sites, synonymous nucleotide diversity (π_s_) and nonsynonymous nucleotide diversity (π_a_) were calculated using *DnaSP *version 5 [77]. The significance of comparison among diversity indexes were accessed by a two sample z-test for comparison between means [78]. *fru *C3 exon and I3 intron haplotype networks were inferred by *TCS v 1.21 *software [79] using statistical parsimony with a 95% connection significance [80], and was manually converted to Newick tree format for some of the neutrality tests. Because recombination interferes with phylogenetic inferences, we performed three different methods to detect recombination events: *GENECONV *[81] and *RDP *both implemented in *RDP *version 3b14 [82]. Tajima´s *D *[30], Fu and Li´s *D *and *F *[31] and Fay and Wu's *H *[32] neutrality tests were performed in *DnaSP *version 5 [77] using a sequence from *Drosophila melanogaster *(GenBank access number: D84437.1) as an outgroup for the *fru *C3 exon analysis and from *Ceratitis capitata *(GenBank access number: AF124047.1) for intron analyses. We used different outgroup sequences because the *C. capitata *sequence available on GenBank included only a small portion of the C3 exon, and the *D. melanogaster *intron showed higher divergence in relation to the sequences obtained in this work, and, as a consequence, its correct alignment was impaired.

One test commonly used to analyze populational data is the McDonnald-Kreitman's test [36], which contrasts fixation and polymorphism levels of synonymous and non-synonymous substitutions for two species in a contingency table [36]. Our data cannot be subjected to McDonnald-Kreitman test since there is no fixed interspecific variation for any of the species considered. We may, however, use a modification of the test proposed by Templeton [37] which implements the contrast of synonymous and nonsynonymous substitutions in tip and interior haplotypes (populational equivalents to the young and old haplotypes contrast of McDonnald-Kreitman test, respectively). Under neutrality synonymous and nonsynonymous substitutions are expected to occur in a same rate both in tip and interior haplotypes, whereas at purifying or positive selection such rate are biased toward synonymous or nonsynonymous substitutions, respectively. We propose here an alternative test of selection which evaluates whether synonymous substitutions have a greater probability of surviving in the population when compared to nonsynonymous substitutions. In a neutrally evolving region, we expect that the probabilities of survival of synonymous and nonsynonymous substitutions through time should come from the same probability distribution. On the other hand, if the region is under purifying selection, we expect that nonsynonymous substitutions have a higher chance of being eliminated [83], whereas if the region is under positive selection, we would find several advantageous nonsynonymous substitutions with higher probability of surviving and spreading in the population [54]. In order to detect this pattern in selection signals, we evaluate the number of haplotypes that derived from each mutation directly from a haplotype network. Because recent mutations, such as those present as singletons or doubletons in the tips of the haplotype network, may not yet have passed through the evolutionary test of survival and reproduction over time [37] and are more affected by drift and chance, we only consider mutations present in at least three haplotypes. To contrast the differential survival probabilities of synonymous and nonsynonymous substitutions in the population, we ranked internal synonymous and nonsynonymous mutations in the haplotype network according to the number of descendent haplotypes derived from them and calculate the difference in synonymous and nonsynonymous ranks summation (R1 and R2) in an improved normal approximation to the Mann-Whitney test [84]. If synonymous and nonsynonymous mutations come from the same distribution, we do not expect to see a significant difference in their ranks, i.e., in neutrality, we expect that synonymous and nonsynonymous mutations should have similar probabilities of survival in a population, and therefore, similar ranks. On the other hand, if nonsynonymous mutations have been selected against, we expect that synonymous mutations would be on average older, and therefore have higher ranks than nonsynonymous mutations, and the opposite would be true if the region is under positive selection. Because it has been shown that tests of selection that rely on the comparison of rates of nonsynonymous to synonymous substitutions have limited power when contrasting sequences with little differentiation [29,47], this test is adequate to look for patterns of selection at the population level by using information derived from the phylogenetic relationships amongst the haplotypes.

## Authors' contributions

ISSJ and RAB designed the experiments. ISSJ collected the data. ISSJ and RAB analyzed the data and wrote the manuscript. All authors read and approved the final manuscript.
